# Clinical utility of combined serum IL-6, PCT, and SAA assays for early diagnosis and severity stratification of neonatal sepsis

**DOI:** 10.3389/fmed.2026.1891357

**Published:** 2026-07-28

**Authors:** Yu Jiang, Juan Yang, Shanshan Yang

**Affiliations:** Department of Neonatology, Affiliated Huai’an Hospital of Xuzhou Medical University, Huai’an, Jiangsu, China

**Keywords:** combined detection, early diagnosis, interleukin 6, neonatal sepsis, procalcitonin, serum amyloid A, severity assessment

## Abstract

**Aim:**

Neonatal sepsis continues to be a leading cause of death and disability in newborns worldwide. Early identification is difficult due to vague and overlapping clinical signs. Reliance on a single biomarker often yields suboptimal sensitivity or specificity. This study aimed to assess the added clinical value of combining serum interleukin-6 (IL-6), procalcitonin (PCT), and serum amyloid A (SAA) for the early recognition and severity grading of neonatal sepsis.

**Methods:**

A total of 140 neonates admitted to the neonatal intensive care unit (NICU) between January 2022 and December 2023 were enrolled. Based on clinical criteria and blood culture results, they were assigned to three groups: confirmed sepsis (*n* = 60), suspected sepsis (*n* = 40), and non-infective control (*n* = 40). Among sepsis patients, 24 were classified as severe and 36 as mild/moderate according to predefined severity criteria. Serum concentrations of IL-6, PCT, and SAA were measured at admission (prior to antibiotic therapy). Diagnostic accuracy was evaluated using receiver operating characteristic (ROC) curves, and associations with disease severity were analyzed.

**Results:**

Serum levels of IL-6, PCT, and SAA were markedly higher in the confirmed sepsis group than in the suspected sepsis and control groups (all *p* < 0.001). Levels were also significantly elevated in severe sepsis compared with mild/moderate cases (*p* < 0.01 for each marker). For diagnosing confirmed sepsis, the area under the ROC curve (AUC) was 0.908 for IL-6, 0.932 for PCT, 0.913 for SAA, and 0.985 for the combined panel (IL-6 + PCT + SAA). The combined approach achieved a sensitivity of 93.3% and specificity of 92.5%, outperforming any single biomarker. Furthermore, all three markers showed positive correlations with severity scores (*p* < 0.01). Optimal cut-off values for diagnosing sepsis were IL-6 > 52.8 pg./mL, PCT > 2.15 ng/mL, and SAA > 28.6 mg/L.

**Conclusion:**

Simultaneous measurement of serum IL-6, PCT, and SAA substantially enhances early diagnostic accuracy for neonatal sepsis and helps differentiate disease severity. This panel can serve as a rapid, reliable, and minimally invasive adjunct to blood culture in the NICU setting.

## Introduction

Neonatal sepsis is a systemic inflammatory response syndrome triggered by bacterial, viral, or fungal pathogens occurring within the first 28 days after birth (1). It remains a primary contributor to neonatal mortality and long-term neurodevelopmental deficits, particularly in low-resource countries (2). In developed nations, the estimated incidence ranges from 1 to 10 per 1,000 live births, whereas in resource-limited settings it may reach 50–170 per 1,000 live births (3). Despite improvements in intensive care, case-fatality rates range from 10 to 30%, with even higher rates among preterm or very low birth weight infants (4).

The clinical presentation of neonatal sepsis is notoriously nonspecific. Early signs such as temperature instability, lethargy, poor feeding, respiratory distress, or irritability can easily be mistaken for non-infectious conditions or physiological adaptations (5). Consequently, clinicians often face a dilemma: delaying antibiotic therapy may lead to rapid progression to septic shock and death, while unnecessary antibiotic exposure promotes antimicrobial resistance, alters the neonatal microbiome, and increases the risk of necrotizing enterocolitis and late-onset sepsis (6).

Blood culture remains the reference standard for confirming neonatal sepsis (7). However, it has several inherent limitations. First, blood culture requires at least 24–48 h to yield a positive result, and in many cases, it may take up to 72 h, which is too late for early decision-making. Second, the sensitivity of a single blood culture in neonates is only about 50–70%, partly because of small blood volume collected and intermittent bacteremia. Third, maternal antibiotic treatment prior to delivery can further reduce culture positivity. Fourth, contamination rates are non-negligible, leading to false-positive results. Given these drawbacks, there is an urgent need for rapid, reliable, and cost-effective biomarkers that can aid in early diagnosis and guide antibiotic stewardship (8).

Interleukin-6 (IL-6) is a pro-inflammatory cytokine that rises rapidly within hours of bacterial infection. It has a short half-life and normalizes quickly after infection control, making it useful for early detection (9). Procalcitonin (PCT) is a precursor of calcitonin that is specifically elevated during bacterial infections but suppressed during viral infections or inflammatory responses. It has been extensively studied in neonatal sepsis and shows good diagnostic accuracy (10). Serum amyloid A (SAA) is an acute-phase protein synthesized mainly by the liver in response to inflammatory stimuli. It rises earlier and more dramatically than C-reactive protein (CRP) and has shown promise in neonatal infection (11).

Each single biomarker, however, has limitations. IL-6 may be elevated in non-infectious inflammatory conditions such as birth asphyxia or meconium aspiration. PCT can be physiologically elevated in healthy newborns during the first 48 h of life. SAA lacks specificity for bacterial versus viral infection. Combining multiple biomarkers that reflect different pathophysiological pathways may enhance diagnostic performance, improve early detection, and help assess disease severity.

The objectives of this study were: (1) to compare serum IL-6, PCT, and SAA levels among confirmed sepsis, suspected sepsis, and non-infective control neonates; (2) to evaluate the diagnostic value of each biomarker alone and in combination for early diagnosis of neonatal sepsis; (3) to identify optimal cut-off values; and (4) to explore the relationship between these biomarkers and sepsis severity.

## Methods

### Study design and setting

This prospective observational study was conducted at our hospital, between January 2022 and December 2023. The study protocol was approved by the Institutional Ethics Committee of our hospital. Written informed consent was obtained from the parents or legal guardians of all participating neonates.

### Participants

A total of 140 neonates admitted to the neonatal intensive care unit (NICU) during the study period were enrolled. They were classified into three groups based on clinical presentation, laboratory findings, and blood culture results.

Inclusion criteria: Neonates of any gestational age admitted within the first 28 days of life with clinical signs suggestive of sepsis (temperature instability, lethargy, poor feeding, apnea, respiratory distress, tachycardia or bradycardia, hypotonia, or poor perfusion) or high-risk factors (maternal chorioamnionitis, prolonged rupture of membranes >18 h, preterm labor, or suspected maternal infection).

Exclusion criteria: (1) major congenital anomalies or chromosomal disorders; (2) perinatal asphyxia requiring therapeutic hypothermia; (3) congenital metabolic diseases; (4) neonates whose parents refused consent; (5) received intravenous antibiotics for >24 h before admission (except intrapartum antibiotic prophylaxis). Neonates with perinatal asphyxia requiring therapeutic hypothermia were specifically excluded because hypoxic–ischemic injury itself can substantially elevate IL-6 and SAA independent of infection; including this population would have confounded the assessment of the biomarker panel’s diagnostic accuracy for sepsis. Consequently, the diagnostic performance of this panel among asphyxiated neonates undergoing therapeutic hypothermia could not be evaluated in the present cohort and remains to be determined in future studies.

Group definitions: Confirmed sepsis group (*n* = 60): Neonates with clinical signs of sepsis AND a positive blood culture for a pathogenic bacterium. Contaminants (coagulase-negative staphylococci, diphtheroids) were excluded unless two separate cultures were positive. Suspected sepsis group (*n* = 40): Neonates with strong clinical signs of sepsis but negative blood culture, who received a full course of antibiotics (≥7 days) and had laboratory evidence of infection (abnormal white blood cell count, immature/total neutrophil ratio >0.2, or elevated CRP > 10 mg/L) that responded to antibiotics. Non-infective control group (*n* = 40): Neonates hospitalized for non-infective conditions (e.g., physiological jaundice, mild transient tachypnea of the newborn, feeding intolerance without infection) with no clinical or laboratory evidence of infection and no antibiotic treatment.

### Severity classification

Among the 60 confirmed sepsis patients, disease severity was categorized according to the modified neonatal sepsis criteria adapted from the International Pediatric Sepsis Consensus Conference:

Mild/moderate sepsis (*n* = 36): Sepsis without organ dysfunction or with only mild transient dysfunction (e.g., mild respiratory support, transient feeding intolerance) that resolved within 24 h.

Severe sepsis (*n* = 24): Sepsis with cardiovascular dysfunction (need for vasoactive agents), acute respiratory distress syndrome requiring mechanical ventilation, renal dysfunction (oliguria, doubling of creatinine), hepatic dysfunction, or disseminated intravascular coagulation.

### Sample collection and biomarker measurement

Peripheral venous blood (2 mL) was drawn from each neonate immediately upon admission, before any antibiotic administration. The sample was divided: 0.5 mL was sent for blood culture using an automated BACTEC system (BD, United States), and the remaining 1.5 mL was centrifuged at 3,000 rpm for 10 min to separate serum. Serum samples were stored at −80 °C until analysis.

IL-6: Measured by electrochemiluminescence immunoassay (Cobas e411 analyzer, Roche Diagnostics, Germany). The detection limit was 1.5 pg./mL; intra-assay and inter-assay coefficients of variation (CV) were <5 and <8%, respectively.

PCT: Measured by an immunofluorescent assay (VIDAS Brahms PCT, bioMérieux, France). The analytical sensitivity was 0.05 ng/mL; CV < 10%.

SAA: Measured by particle-enhanced immunoturbidimetric assay (TBA-2000FR, Toshiba, Japan). The detection range was 3–500 mg/L; CV < 6%.

All laboratory personnel were blinded to the clinical classification.

### Data collection

We collected demographic and clinical information, such as gestational age, birth weight, sex, delivery method, Apgar scores, age at hospital admission, sepsis risk factors (including premature rupture of membranes, maternal fever, and chorioamnionitis), presenting clinical signs, antibiotic treatment duration, length of NICU stay, and patient outcomes (survival or death). For infants diagnosed with sepsis, severity scores—specifically the Neonatal Sequential Organ Failure Assessment (nSOFA)—were also computed.

### Statistical analysis

All statistical analyses were carried out using SPSS version 26.0 (IBM Corp., Armonk, NY, United States) and MedCalc version 20.0 (Ostend, Belgium). The Shapiro–Wilk test was applied to assess the normality of continuous variables. Variables following a normal distribution are presented as mean ± standard deviation (SD) and were compared using one-way ANOVA followed by Tukey’s *post hoc* test. For non-normally distributed variables, data are expressed as median with interquartile range (IQR), and group comparisons were performed using the Kruskal–Wallis H test, followed by Mann–Whitney U tests with Bonferroni correction. Categorical variables were analyzed using the chi-square (*χ*^2^) test or Fisher’s exact test, as appropriate.

The ROC curve analysis was used to assess diagnostic performance. For each biomarker individually and for a combined logistic regression model (IL-6 + PCT + SAA), we derived the AUC, sensitivity, specificity, positive predictive value (PPV), negative predictive value (NPV), and Youden index. Optimal cutoff values were selected by maximizing the Youden index. Spearman’s rank correlation coefficient was employed to evaluate the association between each biomarker and disease severity (nSOFA score).

All statistical tests were two-tailed, and a *p*-value < 0.05 was considered statistically significant.

## Results

### Baseline characteristics of the study population

A total of 140 neonates were enrolled. Baseline characteristics of the three groups are summarized in Table 1. No statistically significant differences were observed among groups regarding gestational age, birth weight, sex, or mode of delivery (all *p* > 0.05). However, the confirmed sepsis group had a significantly lower 5-min Apgar score compared with controls (*p* < 0.05). The most common pathogens isolated from blood cultures were *Klebsiella pneumoniae* (28.3%), *Escherichia coli* (25.0%), *Staphylococcus aureus* (16.7%), Group B Streptococcus (13.3%), and Enterococcus species (8.3%). Five patients (8.3%) had culture-negative but clinically confirmed sepsis based on strong clinical criteria and laboratory response to antibiotics.

**Table 1:** Baseline characteristics of the three groups.

Characteristic	Confirmed sepsis (*n* = 60)	Suspected sepsis (*n* = 40)	Control (*n* = 40)	*P*-value
Gestational age (weeks, mean ± SD)	36.2 ± 2.8	36.8 ± 2.5	37.2 ± 2.3	0.162
Birth weight (g, mean ± SD)	2,850 ± 520	2,910 ± 480	2,980 ± 450	0.381
Male, *n* (%)	34 (56.7)	22 (55.0)	21 (52.5)	0.874
Cesarean section, *n* (%)	28 (46.7)	18 (45.0)	19 (47.5)	0.957
Apgar 5 min < 7, *n* (%)	15 (25.0)	6 (15.0)	4 (10.0)	0.045
Age at admission (days, median [IQR])	5 (2–12)	6 (3–14)	4 (2–8)	0.268
Maternal PROM >18 h, *n* (%)	22 (36.7)	10 (25.0)	6 (15.0)	0.038
NICU stay (days, median [IQR])	18 (12–28)	12 (8–18)	5 (3–9)	<0.001
Mortality, *n* (%)	6 (10.0)	0 (0)	0 (0)	0.014

PROM, premature rupture of membranes; NICU, neonatal intensive care unit.

### Serum levels of IL-6, PCT, and SAA

**Figure 1:**
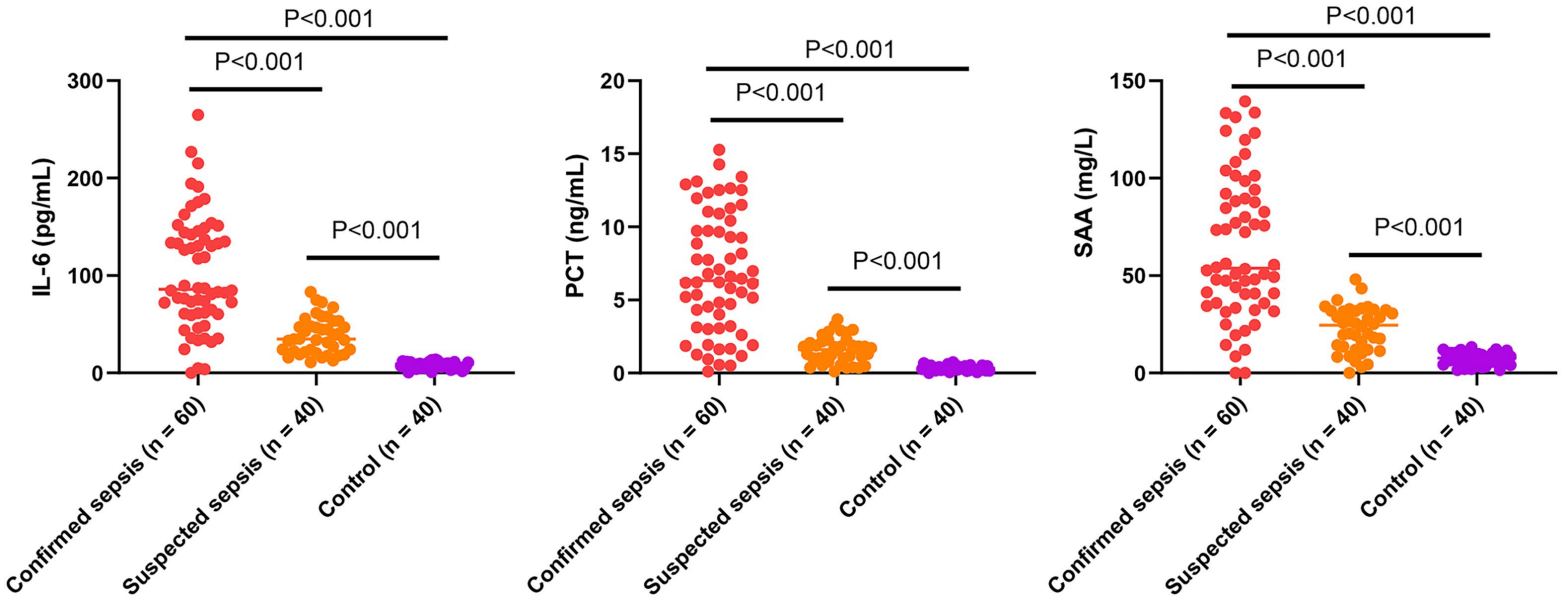
Serum levels of IL 6, PCT, and SAA in the three groups.

Figure 1 present the serum biomarker levels in the three groups. All three biomarkers were significantly higher in the confirmed sepsis group relative to both the suspected sepsis and control groups (*p* < 0.001 for each comparison). The suspected sepsis group also had notably higher levels than the control group (p < 0.001 for each comparison).

**Figure 2:**
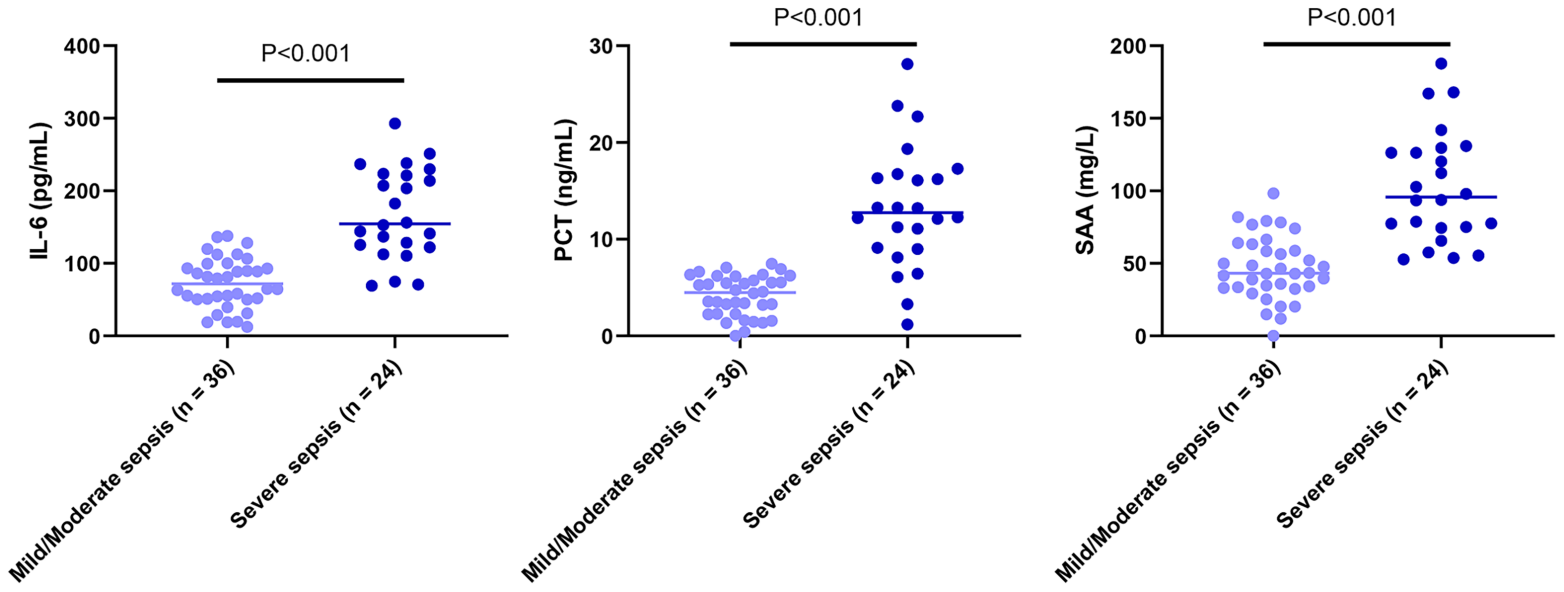
Serum levels of IL 6, PCT, and SAA between the mild/moderate sepsis and severe sepsis groups.

Within the confirmed sepsis group, severe sepsis patients had significantly higher levels of IL-6, PCT, and SAA than mild/moderate sepsis patients (all *p* < 0.001) (Figure 2).

### Correlation between biomarkers and disease severity

**Figure 3:**
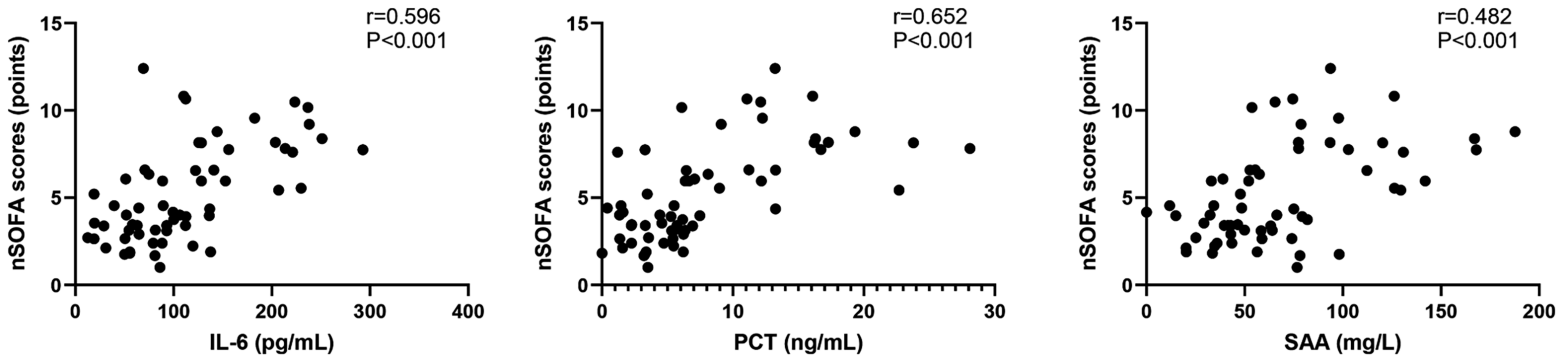
Correlation between serum levels of IL 6, PCT, and SAA and nSOFA scores.

Among confirmed sepsis patients, Spearman correlation analysis demonstrated significant positive correlations between each biomarker and the nSOFA score: IL-6 (*r* = 0.596, *p* < 0.001), PCT (*r* = 0.652, *p* < 0.001), and SAA (*r* = 0.482, *p* < 0.001) (Figure 3).

### Diagnostic performance of individual biomarkers and combined model

**Figure 4:**
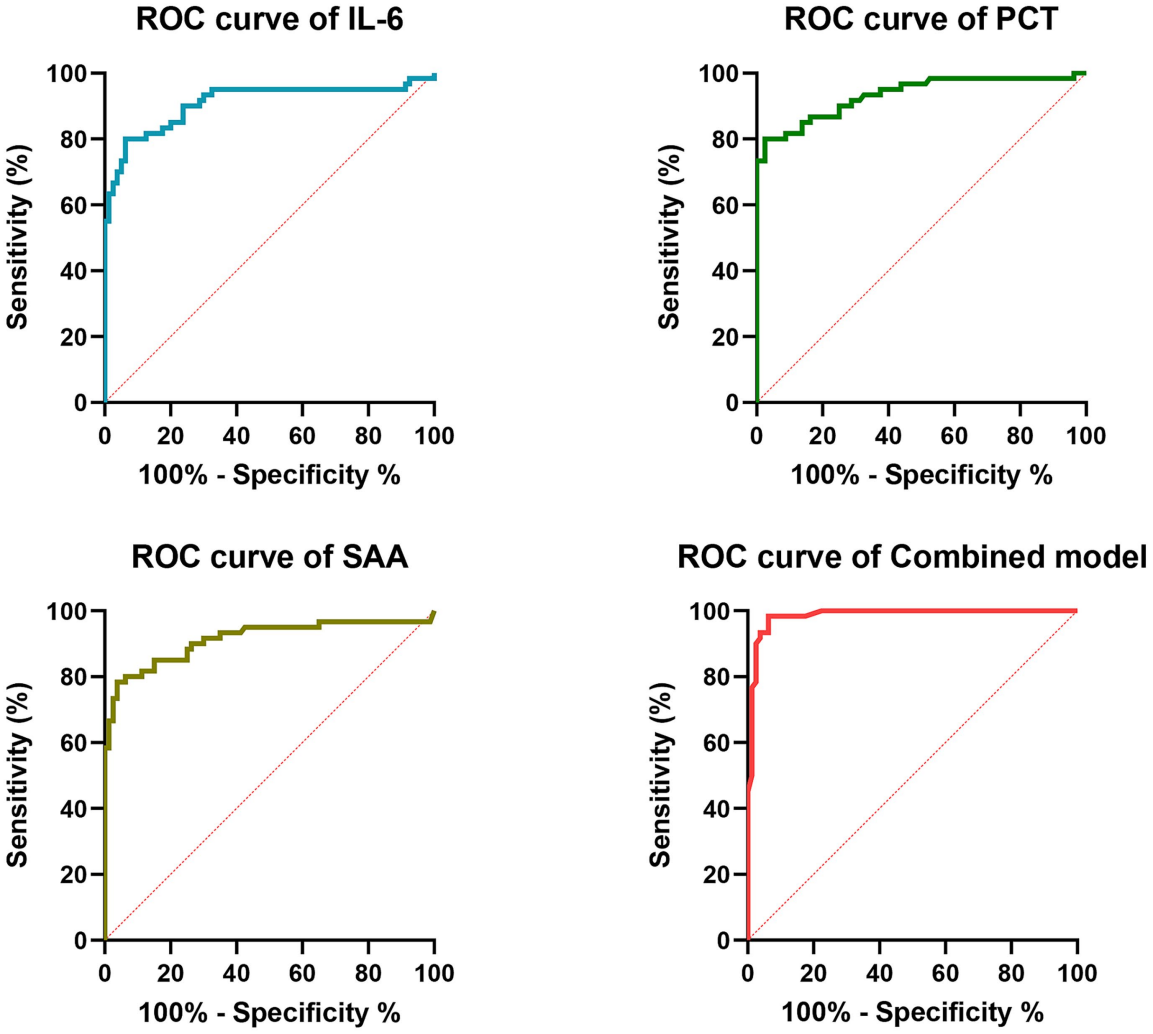
ROC curves for IL-6, PCT, SAA, and combined model.

ROC curve analysis was performed to assess the ability of each biomarker and their combination to differentiate confirmed sepsis from non-sepsis (suspected + control groups). The results are shown in Table 2 and Figure 4.

**Table 2:** ROC analysis for diagnosis of confirmed neonatal sepsis.

Biomarker	AUC (95% CI)	Cut-off value	Sensitivity (%)	Specificity (%)	Youden index
IL-6	0.908 (0.850–0.965)	>52.8 pg./mL	85.0	83.8	0.688
PCT	0.932 (0.888–0.977)	>2.15 ng/mL	88.3	86.3	0.746
SAA	0.913 (0.857–0.968)	>28.6 mg/L	81.7	81.3	0.630
Combined model	0.985 (0.969–1.000)	>0.52*	93.3	92.5	0.858

Combined model probability cut-off derived from logistic regression; AUC, area under the curve; CI, confidence interval. The asterisk denotes the combined-model probability cut-off derived from logistic regression.

All three biomarkers had good diagnostic accuracy (AUC > 0.85). PCT showed the highest individual AUC (0.932), followed by IL-6 (0.908) and SAA (0.913). The combined logistic regression model (IL-6 + PCT + SAA) significantly improved diagnostic performance, achieving an AUC of 0.985 (95% CI: 0.969–1.000). The sensitivity, specificity, PPV, and NPV of the combined model were 93.3, 92.5, 89.7, and 95.2%, respectively, which were superior to any single biomarker.

## Discussion

Neonatal sepsis remains a diagnostic challenge in the NICU due to its subtle and nonspecific early manifestations. The overreliance on blood culture, despite its “gold standard” status, often leads to delayed or missed diagnosis (12). In this prospective study, we demonstrated that the combined measurement of serum IL-6, PCT, and SAA significantly improves early diagnostic accuracy for neonatal sepsis and correlates well with disease severity. Our findings support the use of this biomarker panel as a rapid, non-invasive adjunct to blood culture.

Among the three individual biomarkers, PCT exhibited the highest diagnostic accuracy with an AUC of 0.932, sensitivity of 88.3%, and specificity of 86.3% at a cut-off of 2.15 ng/mL. This is consistent with previous meta-analyses that have reported PCT as one of the most reliable biomarkers for early-onset neonatal sepsis (13, 14). PCT has the advantage of rising specifically in response to bacterial infection, with relatively little elevation during viral infections or non-infectious inflammation (15). However, physiological PCT elevation in the first 48 h of life can complicate interpretation in very early-onset sepsis (16). In our study, the median age at admission was 5–6 days in sepsis groups, partially mitigating this issue.

IL-6 also performed well (AUC 0.908), with a cut-off of 52.8 pg./mL. IL-6 is known to rise within 2–4 h of bacterial invasion, earlier than PCT or CRP, making it particularly valuable for very early diagnosis (17). Its short half-life (approximately 2–4 h) also makes it useful for monitoring treatment response, although we did not assess serial measurements in this study. One limitation of IL-6 is its lack of specificity; it can be elevated in non-infectious conditions like birth asphyxia, meconium aspiration syndrome, or maternal chorioamnionitis without fetal infection (18). Nevertheless, in the appropriate clinical context, a markedly elevated IL-6 level strongly supports bacterial sepsis.

SAA showed slightly lower but still good diagnostic performance (AUC 0.913). SAA is an acute-phase protein that responds more rapidly and dramatically than CRP, with levels increasing up to 1,000-fold within 24 h of infection (19). In our cohort, a cut-off of 28.6 mg/L gave a sensitivity of 81.7% and specificity of 81.3%. However, SAA shares the limitation of poor specificity for bacterial versus viral or fungal infections, and it can also be elevated in non-infectious inflammatory states (20). Despite this, SAA may be particularly useful in resource-limited settings where PCT assays are not available, as SAA can be measured using simple immunoturbidimetric methods.

Our study’s key finding is that the IL-6, PCT, and SAA panel performed substantially better than any individual biomarker, yielding an AUC of 0.985, a sensitivity of 93.3%, and a specificity of 92.5%. This nearly ideal diagnostic accuracy indicates that the three markers reflect complementary facets of the host’s response to bacterial infection. The pathophysiological rationale for this synergy is as follows: IL-6 is an early pro-inflammatory cytokine that initiates the acute-phase response; PCT is a bacterial-specific biomarker that is relatively stable and less affected by non-infectious inflammation; and SAA is a downstream acute-phase protein that amplifies the inflammatory signal. By combining an early cytokine (IL-6), a specific bacterial marker (PCT), and a highly sensitive acute-phase reactant (SAA), the panel compensates for the individual weaknesses of each marker. For example, a neonate with sterile inflammation (e.g., meconium aspiration) might have elevated IL-6 but normal PCT and SAA; such a pattern would reduce false-positive diagnoses. Conversely, a neonate with early-stage sepsis might have normal PCT but elevated IL-6 and SAA, allowing earlier detection before PCT rises. Our results are in line with several recent studies that have advocated for multi-biomarker approaches. A study by An et al. found that the AUC, specificity and sensitivity of the combined detection were better than those of the single detection for neonatal sepsis (21). Our study adds SAA to the panel and confirms that a three-marker combination achieves even higher specificity, reducing unnecessary antibiotic exposure.

All three biomarkers showed significant positive correlations with the nSOFA score, a validated severity scoring system for neonatal sepsis (22). Severe sepsis patients had significantly higher levels of IL-6, PCT, and SAA compared to mild/moderate sepsis patients. The strongest correlation was discovered for PCT, followed by IL-6 and SAA. These findings indicate that biomarker levels not only help diagnose sepsis but also provide prognostic information.

The clinical implications are substantial. A neonate presenting with an extremely elevated PCT (>10 ng/mL) or IL-6 (>200 pg./mL) is likely to have severe sepsis or septic shock and may require immediate aggressive resuscitation, vasoactive support, and broad-spectrum antibiotics. Conversely, borderline elevations (e.g., PCT 2–5 ng/mL) in a stable infant might allow for more targeted therapy and earlier de-escalation. Serial biomarker measurements could further guide the duration of antibiotic therapy, a critical aspect of antimicrobial stewardship in the NICU.

We did not measure CRP in this study, which may be seen as a limitation. CRP remains the most widely used acute-phase protein in many NICUs due to its low cost and wide availability. However, CRP has well-known drawbacks: it rises slowly (12–24 h after infection onset), peaks late, and has poor sensitivity (approximately 60–70%) in the first 24–48 h of life (23). In contrast, IL-6 and PCT rise within 4–6 h, and SAA rises within 8–12 h. Several studies have directly compared these biomarkers and consistently found L-6 and SAA to be superior to CRP for early diagnosis of neonatal sepsis (24). Therefore, while CRP remains a useful confirmatory test, it should not be relied upon for initial decision-making. Our panel offers a faster and more accurate alternative, particularly when combined with clinical assessment.

### Limitations and future directions

This study has several limitations that should be acknowledged. First, it was conducted at a single center with a relatively modest sample size, especially for the severe sepsis subgroup (*n* = 24). Although the results were statistically robust, multi-center validation with larger cohorts is needed to confirm the optimal cut-off values and generalizability. Second, we did not perform serial biomarker measurements. Monitoring the kinetics of IL-6, PCT, and SAA over time could provide additional information on treatment response, prognosis, and optimal antibiotic duration. Third, the control group included only non-infective conditions; we did not include neonates with viral or fungal sepsis, so the specificity of the panel for bacterial versus non-bacterial infections requires further investigation. Fourth, we excluded neonates who received antibiotics before admission, which may have selected a healthier subset of sepsis patients. In real-world practice, many neonates receive intrapartum or early postnatal antibiotics, which could lower biomarker levels and reduce diagnostic accuracy. Fifth, the “suspected sepsis” group, by definition, had negative blood cultures but strong clinical and laboratory evidence of infection. While this reflects clinical reality, it introduces some ambiguity because some of these patients might have had true sepsis missed by culture. Sixth, the study population spanned a clinically heterogeneous age range at admission (2–14 days), encompassing both early-onset-type and late-onset-type presentations, for which the causative pathogens and disease trajectories may differ; given the limited overall sample size, and particularly the small severe sepsis subgroup (*n* = 24), we were unable to perform a statistically robust age-stratified subgroup analysis, and this issue should be addressed in future, larger multicenter cohorts. Lastly, a formal cost-effectiveness analysis was not carried out. Although IL-6, PCT, and SAA assays are more expensive than CRP, the potential savings from reduced unnecessary antibiotic use, shorter hospital stays, and fewer adverse outcomes may justify their use in high-risk NICU populations.

Future studies should aim on: (1) confirm our results in a larger, multicenter cohort that includes preterm and very low birth weight infants; (2) examine the utility of repeated biomarker measurements for guiding antibiotic cessation (i.e., antimicrobial stewardship protocols); (3) create a rapid bedside point-of-care test capable of concurrently detecting IL-6, PCT, and SAA from a small blood sample (e.g., 100 μL) with a turnaround time under 30 min; (4) explore whether this panel can differentiate between early-onset and late-onset sepsis, given their distinct pathophysiologies and microbial causes; and (5) determine how biomarker-guided algorithms affect clinical endpoints such as mortality, NICU length of stay, and neurodevelopmental outcomes.

### Clinical implications

Notwithstanding these constraints, our results offer several real-world applications. In the NICU setting, we propose the following algorithm for neonates with suspected sepsis:

Immediate collection of blood culture and serum for IL-6, PCT, and SAA before antibiotic administration.

If the combined panel score exceeds the cut-off (probability >0.52) or if any two markers are markedly elevated, initiate broad-spectrum antibiotics without delay.

If the panel is negative (all three markers below cut-offs) and the clinical suspicion is low, consider close observation without antibiotics or a short course pending culture results.

For confirmed sepsis, use the initial biomarker levels to assess severity: PCT > 10 ng/mL or IL-6 > 200 pg./mL should prompt consideration of severe sepsis/septic shock and escalation of care.

Serial measurements every 24–48 h can be used to monitor treatment response; a ≥ 80% decline in IL-6 or PCT by day 3 suggests adequate therapy and supports early de-escalation.

Such an approach could reduce the time to appropriate therapy, decrease unnecessary antibiotic exposure, and improve outcomes in neonatal sepsis.

## Conclusion

In conclusion, this study demonstrates that the simultaneous measurement of serum IL-6, PCT, and SAA provides excellent diagnostic accuracy for early neonatal sepsis and correlates significantly with disease severity. The three-marker panel outperforms any single biomarker, offering high sensitivity (93.3%) and specificity (92.5%). This non-invasive, rapid test can serve as a valuable adjunct to blood culture in the NICU, facilitating earlier diagnosis, risk stratification, and potentially guiding antibiotic stewardship. To confirm these findings and develop standardized protocols for clinical use, larger multi-center studies are needed.

## Data Availability

The original contributions presented in the study are included in the article/supplementary material, further inquiries can be directed to the corresponding author.
